# Safety and Long-Term Efficacy Outcomes for Endovascular Treatment of Wide-Neck Bifurcation Aneurysms of the Middle Cerebral Artery: Insights From the SMART Registry

**DOI:** 10.3389/fneur.2022.830296

**Published:** 2022-02-07

**Authors:** Reade De Leacy, Devin V. Bageac, Neha Siddiqui, Richard J. Bellon, Min S. Park, Clemens M. Schirmer, Keith B. Woodward, Osama O. Zaidat, Alejandro M. Spiotta

**Affiliations:** ^1^Department of Neurosurgery, Mount Sinai Hospital, New York, NY, United States; ^2^Radiology Imaging Associates PC, Englewood, CO, United States; ^3^Department of Neurosurgery, University of Virginia, Charlottesville, VA, United States; ^4^Department of Neurosurgery and Neuroscience Institute, Geisinger Health System, Wilkes-Barre, PA, United States; ^5^Research Institute of Neurointervention, Paracelsus Medical University, Salzburg, Austria; ^6^Vista Radiology, Knoxville, TN, United States; ^7^Mercy Health, Toledo, OH, United States; ^8^Department of Neurosurgery, Medical University of South Carolina, Charleston, SC, United States

**Keywords:** wide-neck aneurysm, bifurcation aneurysm, middle cerebral artery, aneurysm coiling, coiling outcomes

## Abstract

**Introduction:**

Wide-necked middle cerebral artery bifurcation aneurysms pose specific challenges to endovascular management. Surgical clipping remains the standard treatment approach for these aneurysms in many centers. While recent data suggests the endovascular treatment may be comparable, a prospective datapoint has been lacking.

**Materials and Methods:**

The Penumbra SMART registry, a prospective, multi-center, single-arm outcomes registry of Penumbra coil-treated aneurysms, was queried for endovascularly treated MCA bifurcation aneurysms with wide necks (dome:neck ratio <2 or neck >4 mm). Safety and efficacy outcomes were summarized for ruptured and unruptured aneurysms, including rupture, complication rate, and 1-year occlusion outcomes.

**Results:**

Seventy-two aneurysms across 31 sites were enrolled. Of these, a total of 15 presented as ruptured aneurysms. Serious adverse events were reported in 21 (29.2%) of patients, with 8 (11.1%) attributed to the device/procedure. Immediately postoperatively, 75.0% of cases achieved “adequate” Raymond Roy Class I (40.3%) or II (34.7%) occlusion outcomes. Of the 72 patients treated, 60 (83.3%) underwent follow-up angiography at 1 year, and among these, 95.0% had 1-year occlusion outcomes of Raymond Roy Class I (71.7%) or II (23.3%). A total of 6 aneurysms (10.0%) were required or were planned for retreatment at the last follow-up.

**Conclusion:**

This study represents the most significant prospective sample of endovascularly treated wide-neck MCA bifurcation aneurysms conducted to date. It supports the safety and efficacy of endovascular treatment of these aneurysms.

## Introduction

Middle cerebral artery (MCA) bifurcation aneurysms are among the most common intracranial aneurysms. Historically, they have been considered unique challenges to endovascular treatment due to their distal location and frequent incorporation of small-caliber vessels (1). These challenges are amplified in the case of wide-necked MCA bifurcation aneurysms, which often require adjunctive devices such as stents or balloons to achieve acceptable rates of adequate and durable embolization. Unfortunately, studies regarding outcomes following endovascular treatment for these aneurysms have been limited to retrospective series, most of which were conducted before introducing currently available devices for stent-assisted coiling (2–5).

We present results from the Penumbra SMART Registry, which represents the largest prospective sample of endovascularly treated wide-neck MCA bifurcation aneurysms conducted to date. The purpose of this study is to identify the rate of successful occlusion at 1 year and procedural complications following endovascular treatment and present a high-quality prospective datapoint to inform clinical decision-making.

## Materials and Methods

### Data Sources

The Penumbra SMART Coil (Penumbra Inc.; Alameda, CA, United States) is commonly utilized in the treatment of intracranial aneurysms. The SMART registry is a prospective, single-arm, multi-center registry that includes imaging, demographic characteristics, procedural information and clinical outcomes for, patients treated for intracranial aneurysms, and vascular lesions using SMART COIL® (SMART), Penumbra Coil 400® (PC 400), and Penumbra Occlusive Device® (POD) (Penumbra Inc.; Alameda, CA) (6). The Penumbra SMART Registry consisted of an initial 75 sites that were activated, from which 68 enrolled subjects.

### Design and Eligibility

We present a subgroup analysis of patients treated with coiling for wide-neck MCA bifurcation aneurysms within the Penumbra SMART registry. Within the registry, the MCA wide-neck aneurysm cohort consisted of subjects enrolled from 31 sites.

The study was designed in accordance with the relevant aspects of clinical research regulations and received approval from the Institutional Review Board (IRB) and Ethics Committee (EC) from each participating site. Written informed consent was provided by patients or their legally authorized representative (LAR). For patients treated emergently, consent was able to be obtained up to 24 h following the procedure. Penumbra, Inc. sponsored oversight throughout the duration of the trial.

In order to qualify for inclusion, treatment was required to conform to one of the cleared indications for the investigational devices, which includes intracranial aneurysms. In addition, patients were excluded from participation if the investigational devices accounted for <75% of a total number of coils implanted in the target aneurysm, their life expectancy was <1year (excluding poor prognosis due to aneurysm-related illness), or they were participating in another clinical investigation that could confound the evaluation of the registry device. Patients with wide-necked aneurysms (dome:neck ratio <2 or neck > 4 mm) that were present at the MCA bifurcation were included.

### Procedure and Data Collection

**Figure 1:**
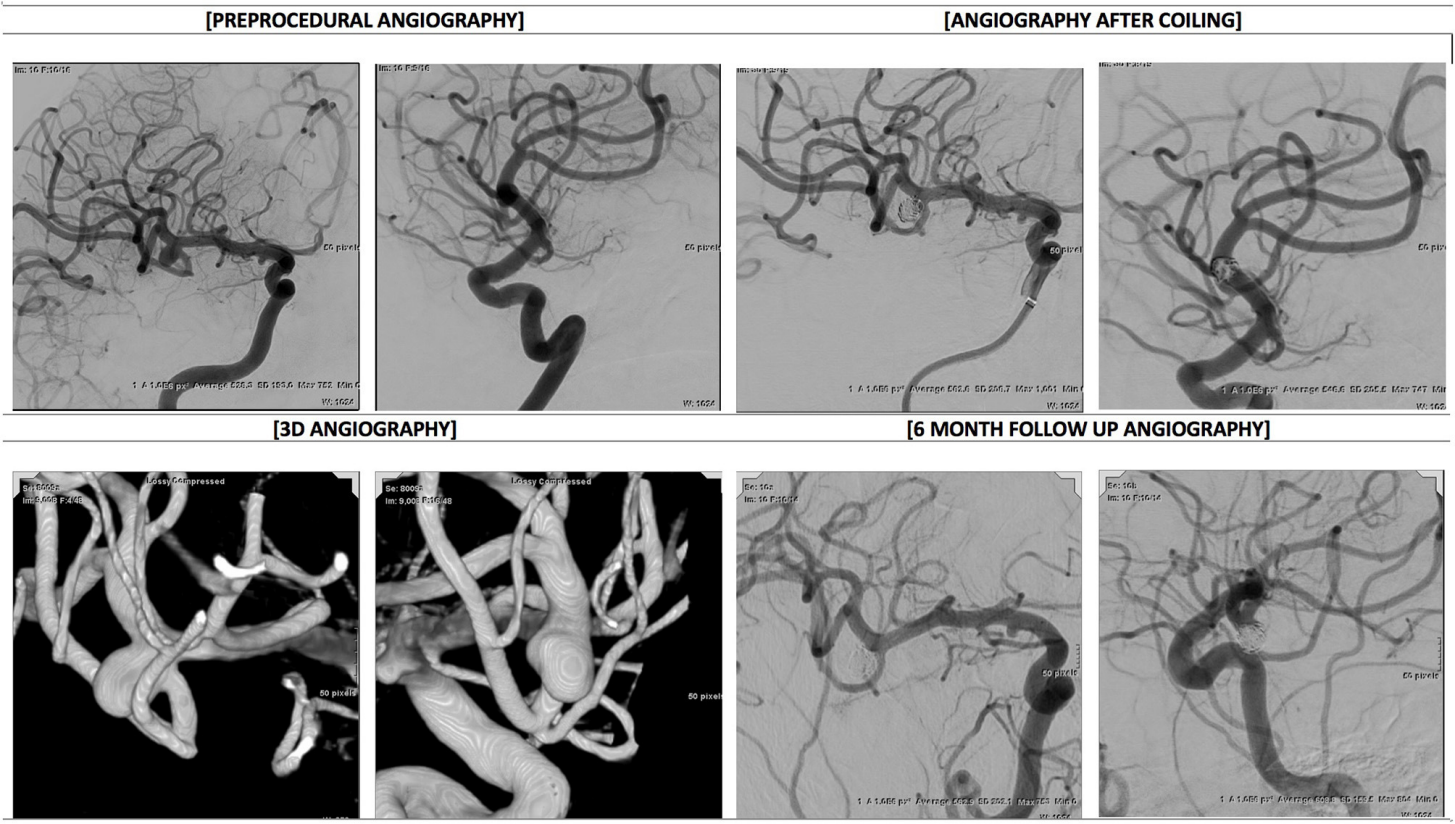
Angiography before and after coiling−53 y.o. male patient presents with unruptured 6 mm right MCA bifurcation aneurysm. Baseline and immediately post stent assisted coiling DSA images in working projections, baseline 3D images in orthogonal projections and final DSA images available at 6 months follow up shows Raymond Roy 2 occlusion with small residual dog ear best depicted on lateral oblique projection (bottom right panel).

Coiling procedures were performed as per routine site standard of care, without restrictions regarding endovascular technique, registry device specifications, or use of adjunctive technologies. The Modified Rankin Scale (mRS) was captured at admission and at 1-year if available per the site standard of care. Cerebral angiograms were obtained immediately post-procedure and at 1-year (±6 months) (Figure 1). At both angiographic timepoints, the aneurysm occlusion status was determined by the treating physician. Demographics, medical history, procedural, angiographic, and adverse event (AE) data were collected. AEs related to the procedure or device and all serious adverse events (SAEs) were collected from the time of enrollment through registry exit. Safety data were reviewed by centralized monitors to ensure accurate event reporting.

### Study Definitions

Wide-necked aneurysms were defined as those with dome-to-neck ratio <2 or neck width >4 mm. Aneurysm occlusion status was graded angiographically according to the Raymond–Roy Occlusion Classification (RROC), wherein Class I represents complete occlusion, Class II represents a residual neck, and Class III is a residual aneurysm. The severity of ruptured aneurysms at admission was determined by the Hunt and Hess (H&H) scale.

Periprocedural adverse events (AEs) were defined as events occurring during or within 24 h of the index procedure. Thromboembolic or rupture/perforation-related AEs were recorded regardless of the presence or absence of symptoms. The investigators determined the relationships of an AE to the device and procedure (definite, probable, possible, unrelated). For this analysis, events reported as definite, probable, or possible were considered as related.

### Statistical Analysis

Descriptive statistics were calculated for demographic, procedural, angiographic, and adverse event data, including the number of observations, mean, standard deviation (SD), median, interquartile range (IQR), minimum and maximum for continuous variables, and counts and percentages for discrete variables. In addition, subgroup analysis was conducted based upon rupture status (acutely ruptured vs. remote/unruptured).

## Results

### Demographics and Baseline Characteristics

Between June 2016 and August 2018, the SMART registry enrolled 72 wide-neck MCA bifurcation aneurysms across 31 sites. Over the study period, five patients (6.9%) were lost to follow-up, and one patient (1.4%) withdrew consent, yielding a completion of follow-up rate of 91.7%. Patient demographics and past medical history are summarized in Table 1. The mean patient age was 62.4 years [SD 11.47, median 64.5, IQR (54.5, 69.5)], and 50% were over 65. The majority of patients were female (69.4%). Hypertension was present in the past medical history of 68.1% of patients, 77.8% had a history of smoking, and 19.4% had a family history of cerebral aneurysms.

**Table 1:** Demographics and past medical history.

***N* (%) (unless otherwise noted)**	**All subjects (*N* = 72)**	**Ruptured (*N* = 15)**	**Unruptured (*N* = 57)**	***P*-value**
Age (mean, SD)	62.4 (11.47)	60.8 (13.65)	62.9 (10.92)	0.532
Female	50 (69.4%)	12 (80.0%)	38 (66.7%)	0.317
Ethnicity				
Hispanic or latino	4 (5.6%)	1 (6.7%)	3 (5.3%)	0.826
Not hispanic or latino	20 (27.8%)	4 (26.7%)	16 (28.1%)	
Not reported	48 (66.7 %)	10 (66.7%)	38 (66.7%)	
Race				
Black or African American	1 (1.4%)	0 (0.0%)	1 (1.8%)	0.443
White	22 (30.5%)	5 (33.3%)	17 (29.8%)	
Other	1 (1.4%)	0 (0.0%)	1 (1.8%)	
Not reported	48 (66.7 %)	10 (66.7%)	38 (66.7%)	
Past medical history				
Previous stroke	18 (25.0%)	20.0% (3/15)	15 (26.3%)	0.617
Ischemic	7 (9.7%)	6.7% (1/15)	6 (10.5%)	0.653
Hemorrhagic	7 (9.7%)	13.3% (2/15)	5 (8.8%)	0.596
Transient ischemic attack	2 (2.8%)	0 (0.0%)	2 (3.5%)	0.459
Unknown	2 (2.8%)	0 (0.0%)	2 (3.5%)	0.459
Headache	28 (38.9%)	6 (40.0%)	22 (38.6%)	0.920
Migraine	7 (9.7%)	1 (6.7%)	6 (10.5%)	0.653
Traumatic brain injury	2 (2.8%)	0 (0.0%)	2 (3.5%)	0.459
Myocardial infarction	6 (8.3%)	0 (0.0%)	6 (10.5%)	0.190
Hypertension	49 (68.1%)	10 (66.7%)	39 (68.4%)	0.896
Thyroid disorder	13 (18.1%)	1 (6.7%)	12 (21.1%)	0.197
Diabetes	10 (13.9%)	4 (26.7%)	6 (10.5%)	0.107
Family history		9 (60.0%)	34 (59.6%)	0.984
Family history of cerebral aneurysm or vascular malformation	14 (19.4%)	1 (6.7%)	15 (26.3%)	
Polycystic kidney disease	1 (1.4%)	0 (0.0%)	1 (1.8%)	0.603
Fibromuscular dysplasia	0 (0.0%)	0 (0.0%)	0 (0.0%)	>0.999
Social history				
Smoking	56 (77.8%)	13 (86.7%)	43 (75.4%)	0.352
Current	35 (48.6%)	12 (80.0%)	23 (40.4%)	
Former	21 (29.2%)	1 (6.7%)	20 (35.1%)	
Cocaine use	2 (2.8%)	1 (6.7%)	1 (1.8%)	0.303

Aneurysm characteristics are summarized in Table 2. The average maximum aneurysm diameter was 6.8 mm (SD 3.65), 15.3% were >10 mm in size, and none were giant (>25 mm). Saccular aneurysms were found in 86.1% of patients, and fusiform aneurysms were found in 6.9%.

**Table 2:** Aneurysm characteristics.

	**All subjects (*N* = 72)**	**Ruptured (*N* = 15)**	**Unruptured (*N* = 57)**	***P*-value**
Aneurysm Size (mm) (mean, SD)	6.8 (3.65)	9.4 (5.58)	6.1 (2.58)	0.001^**^
<10 mm	84.7% (61/72)	60.0% (9/15)	91.2% (52/57)	0.003^**^
>10 to 25 mm	15.3% (11/72)	40.0% (6/15)	8.8% (5/57)	
Dome to Neck Ratio (mean, SD)	1.64 (0.47)	1.94 (0.68)	1.56 (0.35)	0.004^**^
Neck ≥4 mm	54.2% (39/72)	66.7% (10/15)	50.9% (29/57)	0.276
Aneurysm type				
Saccular	86.1% (62/72)	80.0% (12/15)	87.7% (50/57)	0.441
Fusiform	6.9% (5/72)	6.7% (1/15)	7.0% (4/57)	
Other	6.9% (5/72)	13.3% (2/15)	5.3% (3/57)	
Lesion side				
Right	58.3% (42/72)	46.7% (7/15)	61.4% (35/57)	0.303
Left	40.3% (29/72)	46.7% (7/15)	38.6% (22/57)	
Unknown	1.4% (1/72)	6.7% (1/15)	0.0% (0/57)	
Previously treated with coils	9.7% (7/72)	0.0% (0/15)	12.3% (7/57)	0.153
Acutely ruptured				
Hunt and hess grade I	–	26.7% (4/15)	–	N/A
Grade II	–	26.7% (4/15)	–	
Grade III	–	26.7% (4/15)	–	
Grade IV	–	13.3% (2/15)	–	
Grade V	–	0% (0/0)	–	

** *Significant at alpha = 0.05 level*.

Aneurysms morphology defined as “Other” included three bilobed aneurysms (4.2%), one irregular remnant after the previous coiling (1.4%), and one small ruptured aneurysm for which morphology could not be characterized (1.4%). Fifteen aneurysms (20.8%) had undergone the previous coiling. Acutely ruptured aneurysms accounted for 20.8% (15/72) of the cohort, of which nine (60.1%) presented as H&H Grade I-III, four (26.7%) presented as H&H Grade IV, and two (13.3%) presented as H&H Grade V.

### Procedural Characteristics and Outcomes

Unassisted coiling was used in 43.1% of cases, while the remaining employed adjunctive devices such as stents or balloons (Table 3). Balloon-assisted coiling without stenting was employed in acutely ruptured cases only. Immediate RROC Class I or II occlusion was achieved in 75.0% of cases, with 40.3% of cases achieving Class I occlusion.

**Table 3:** Safety and efficacy outcomes.

	**All Subjects (*N* = 72)**	**Ruptured (*N* = 15)**	**Unruptured (*N* = 57)**	***P*-Value**
Adjunctive technology used during treatment^*^				
Unassisted coiling	31 (43.1%)	9 (60.0%)	22 (38.6%)	0.136
Stent-assisted coiling	32 (44.4%)	2 (13.3%)	30 (52.6%)	0.006^**^
Balloon-assisted coiling	5 (6.9%)	4 (26.7%)	1 (1.8%)	<0.001^**^
Balloon and stent-assisted coiling	4 (5.6%)	0 (0 %)	4 (7.0%)	0.289
Post-procedure RROC				
Class I to II	54 (75.0%)	11 (73.3%)	43 (75.4%)	0.865
Class I	29 (40.3%)	6 (40.0%)	23 (40.4%)	0.984
Class II	25 (34.7%)	5 (33.3%)	20 (35.1%)	
Class III	18 (25.0%)	4 (26.7%)	14 (24.6%)	
1-year RROC				
Class I to II	57 (95.0%)	5 (71.4%)	52 (98.1%)	0.002^**^
Class I	43 (71.7%)	3 (42.9%)	40 (75.5%)	0.007^**^
Class II	14 (23.3%)	2 (28.6%)	12 (22.6%)	
Class III	3 (5.0%)	2 (28.6%)	1 (1.9%)	
1-year change in Raymond-Roy occlusion*, n = 60*				
Better (progressive occlusion)	26 (43.3%)	2 (28.6%)	24 (45.3%)	0.557274
Stable	30 (50.0%)	4 (57.1%)	26 (49.1%)	
Worse (recanalization)	4 (6.7%)	1 (14.3%)	3 (5.7%)	
Retreatment required*, n = 60*	6 (10.0%)	3 (42%)	3 (5.7%)	0 002^**^
Completed during follow-up period	1 (1.7%)	1 (14.3%)	0 (0%)	0.006^**^
Planned for after follow-up period	5 (8.3%)	2 (28.6%)	3 (5.7%)	0.039^**^
Total serious adverse events^*^	21 (29.2%)	8 (53.3%)	13 (22.9%)	0.0206^**^
Intraprocedural or within 24 h	5 (6.9%)	1 (6.7%)	4 (7.0%)	0.962
After 24 h through 365 day	19 (26.4%)	8 (53.3%)	11 (19.3%)	0.008^**^
Device or procedure-related serious adverse events^*^	8 (11.1%)	2 (13.33%)	6 (10.5%)	0.757
Intraprocedural or within 24 h	6 (8.3%)	1 (6.6%)	4 (7.0%)	0.960
After 24 h through 365 day	3 (4.2%)	1 (6.6%)	5 (8.8%)	0.795

* *Data is presented as count of number of patients effected. Some patients experienced both immediate and delayed adverse events*.

** *Significant at alpha = 0.05 level*.

Sixty patients (83%) underwent follow-up angiography at 1 year, with the mean follow-up of all 72 patients being 11 months (SD 2.24 months). Among the 60 patients with 1-year follow-up, RROC Class I or II occlusion had increased to 95.0%, with 72.0% of cases achieving Class I occlusion. Throughout the follow-up period, 43.3% of treated aneurysms demonstrated progressive occlusion, while 7.0% recanalized. Six aneurysms (10.0%) were deemed to require retreatment by the treating interventionist, though only one underwent retreatment during the 1-year follow-up period. The adjunctive materials used for those aneurysms did not significantly differ of those that did not undergo retreatment, with 4 cases using unassisted coiling (*p* = 0.2627), 1 with stent-assisted (*p* = 0.1868) and 1 with balloon-assisted coiling (*p* = 0.3898).

Twenty-one patients (29.2%) within the cohort experienced an SAE, with 53.3% (*n* = 8) of ruptured aneurysms and 22.9% (*n* = 13) of unruptured experiencing a SAE (*p* = 0.0206). Eight SAE (11.1%) were found to be related to the device or procedure. Of these device or procedure-related SAEs, six (8.3%) occurred during or within 24 h of the treatment procedure. These events included two cases of intraprocedural aneurysm rupture, three cases of symptomatic intraprocedural thrombosis, and one case of severe hypotension. There were seven (9.7%) device or procedure-related SAEs that occurred between 1 day and 1 year following the treatment procedure. These included one case of access site bleeding, one case of transient ischemic attack, and one case of ischemic stroke. There was one procedure-related death (1.4%) recorded in the study, which occurred due to intraprocedural thrombosis.

## Discussion

This study presents prospectively collected real-world outcomes following the endovascular treatment of wide-neck MCA bifurcation aneurysms. We observed a self-reported 95.0% rate of adequate occlusion (RROC Class I or II, as validated by previous studies) at one year, with 9.7% of aneurysms requiring or planned for retreatment at 1 year (7, 8). The total rate of device or procedure-related adverse events was 11.1%, of which 75.0% occurred intraprocedural. The rate of self-reported complete occlusion (RROC I) at 1 year was 71.1%.

These results compare favorably to previous studies on the endovascular treatment of wide-neck MCA bifurcation aneurysms. Yan et al. observed a complete occlusion rate of 78.7% in 47 patients at an average follow-up interval of 11.7 months. Adequate occlusion rates at follow-up were not reported (4). In a series of 72 complex MCA aneurysms (51 occurring at the bifurcation), Zhou et al. reported that 93.4% of all aneurysms were completely occluded, improved, or stable, and 4.9% recurred (5). These older studies help to illustrate the technical and technological advancements in the neurointerventional space. In a 2011 series on 48 MCA aneurysms with long term follow-up, Vendrell et al. reported 34 complete occlusions (71%), 14 partial treatments (29%), 7 recurrences (14.6%) occurred, and 5 retreatments (10.4%) (3). Unsurprisingly, our study confirmed that rupture status was significantly associated with retreatment rate (*p* = 0.00208), and final occlusion status (*p* = 0.00236).

MCA bifurcation coiling occlusion rates in our study are comparable to occlusion outcomes for surgically clipped MCA bifurcation aneurysms in other series. A 2013 study of 631 MCA aneurysms by Rodríguez-Hernández et al. found a 98.3% final occlusion rate when employing a “clip first” policy for MCA aneurysms, 90.0% of which were MCA bifurcation aneurysms (9). However, the authors did not specify aneurysm size and included aneurysms that were not wide-necked aneurysms. A study by Marchi et al. summarizing outcomes of clipped MCA bifurcation aneurysms found a final occlusion rate of 94.0% (10). A recent single-site retrospective comparison of a “coil-first” protocol by Pflaeging et al. found a 6-month adequate occlusion rate (RROC1 or 2) of 86.2% for endovascularly coiled aneurysms vs. 96.4% for surgically clipped (11). Our prospective, self-reported findings at a longer follow-up of 1 year demonstrate higher endovascular final occlusion rates that are comparable to the clinical ones.

Our study compares favorably with retreatment and complication rates for coiling and approaches retreatment rates for surgical clipping of MCA bifurcation aneurysms. A 2011 study found a 9.6% retreatment rate for endovascularly treated MCA aneurysms (12). Pfagling et al. found complication rates of 16.7% in surgically clipped MCA bifurcation aneurysms and 20.0% in endovascularly treated aneurysms (11). Previously mentioned studies by Zhou et al. (5) and Vendrell et al. (3) found retreatment rates from 4.9 to 10.4%. The ISAT trial found an initial 17.4% retreatment rate for endovascularly treated aneurysms, which was higher than our findings of 9.7% retreatment rates (13). It also found aneurysm coiling intraprocedural complication rates of 6%, lower than our study of 8.3% for wide neck MCA bifurcation aneurysms. While ISAT was not specific to MCA bifurcation aneurysms or wide-neck aneurysms, a decrease in retreatment rates demonstrates the progress of the endovascular techniques and the devices available to treat intracranial aneurysms, including even traditionally difficult ones such as wide neck MCA bifurcation aneurysms.

Our study, like many prior, does not account for enrolling interventionalist experience. High-volume sites are typically sought after for sponsored prospective research, and this, in turn, may lead to bias in terms of operator experience and patient outcomes. Efforts to overcome this potential bias can be somewhat mitigated in large registries with multiple enrolling centers. The SMART registry involved 65 sites from which 31 submitted wide neck MCA bifurcation aneurysms for treatment underlying the real-world applicability of these data.

By design, the SMART study did not have a comparison arm. Newer wide neck aneurysm bifurcation devices such as the Woven Endobridge (WEB^TM^) (MicroVention, Aliso Viejo, CA, United States) and Pulserider^TM^ aneurysm neck reconstruction device (Cerenovus, Miami, FL, United States), are also indicated for our patient population and may offer an improved safety profile accompanied by high long-term occlusion and low retreatment rates. Results from the core lab adjudicated WEB-IT trial demonstrated adequate occlusion rates of 84.6% for wide-neck bifurcation aneurysms that included but were not exclusive to MCA bifurcation aneurysms. In a retrospective study comparing WEB to coiling-based techniques in the treatment of MCA bifurcation aneurysms, retreatment rates were significantly higher in the WEB group (21.1%) compared with the coiling group with (5.9%) or without (2.2%) stent placement (*P* < 0.05) (2). In this same retrospective study, an occlusion rate of 86.6–97.4%, depending on the technique of stent-assisted coiling vs. coiling vs. WEB, was found. Their follow-up study found a similar 87.1–96.8% (14). This is similar to the finding of 95.0% adequate occlusion at the last follow-up in our prospective analysis. The ANSWER trial studied the Pulserider device in 34 unruptured wide-neck bifurcation aneurysms located at the ICA terminus or basilar apex. MCA bifurcation aneurysms were not included. The authors found an RROC I and II rate of 87.9% at 6-month follow-up (15). The follow-up study to ANSWER, NAPA (NCT03383666) was designed to examine outcomes for wide neck intracranial aneurysm located at a bifurcation and includes MCA lesions. However, this trial was halted prematurely due to slow enrollment (*n* = 21).

Our study found no significant difference between adjunctive technology used in coiling (unassisted vs. stent-assisted vs. balloon-assisted) and need for retreatment. It is possible that alternate methods have different rates of need for retreatment. Flow diversion and adjustable neck bridging meshes represent less common but alternate methods for treating wide neck MCA bifurcation aneurysms (16–19). Early experience with small volume datasets suggests flow diversion results in adequate occlusion rates of 80.0%. However, further larger studies will be required before an expected occlusion rate, and a reliable safety profile can be cited with confidence. Flow diversion at the MCA bifurcation may also lead to remodeling and, in some cases, occlusion of the covered or jailed segment. Although the short and intermediate-term clinical consequences of such flow alteration appear to be negligible based on the limited studies published to date, in principle, efforts to maintain normal branching vascular anatomy supplying the eloquent cortex remain a primary goal. Even less data is available for both the Comaneci (Rapid Medical, Pueblo, CO, United States) and Contour (Cerus Endovascular, Fremont, CA, United States) devices. Each device may show benefit over competing techniques/devices such as balloon assistance or WEB placement, but large volume prospective data allowing direct comparison to those and our results from the SMART dataset is lacking at present (18, 19). Our study finds that many aneurysms were coiled without the use of adjunctive devices, which has been previously been noted in the literature and may potentially due to the increased efficiency in endovascular technique (20, 21).

### Limitations

The primary limitation of this study lies with aneurysm occlusion status being determined by the treating physician and not by a blinded core lab. Results from blinded studies such as BRANCH suggest that without blinding, overreporting of adequate occlusion may be an issue (22). However, this type of bias would likely not affect the reported rate of planned retreatment, which is a more robust metric of treatment failure. Rates of procedural complications are similarly less likely to be affected by this unblinded design. The prospective nature of the study as a whole allows for completeness and uniformity of data collection that stands as a limitation to previous retrospective studies. Furthermore, the outcomes from a single coil type may not generalize to all coils, however, previous studies have demonstrated that coil type potentially may not have a significant impact on angiographic outcomes (23).

## Conclusion

This study presents the largest prospectively collected dataset describing real-world outcomes following the endovascular coiling of wide-neck MCA bifurcation aneurysms. These results support the safety and efficacy of endovascular techniques in treating this challenging aneurysm subtype and will aid the clinical decision-making process in the setting of equipoise between different treatment modalities.

## Data Availability Statement

The raw data supporting the conclusions of this article will be made available by the authors, without undue reservation.

## Ethics Statement

The studies involving human participants were reviewed and approved by the Institutional Review Board (IRB) and Ethics Committee (EC) from each participating site. The full list is available as a Supplementary File. The patients/participants provided their written informed consent to participate in this study.

## Author Contributions

RD and AS: study conception and design. RD, RB, MP, CS, KW, OZ, and AS: data collection. NS, DB, and RD: analysis and interpretation of results. NS, DB, and RD: draft manuscript preparation. All authors reviewed the results and approved the final version of the manuscript.

## Funding

This work was funded by Penumbra, Inc.

## Conflict of Interest

This study received funding from Penumbra, Inc. The funder was involved with the study design, data collection, and statistical analysis. The funder was not involved in the interpretation of data, the writing of this article or the decision to submit it for publication. RD is a consultant for Imperative Care, Stryker Neurovascular, Cerenovus, Penumbra, and has equity in Synchron, Endostream, Q'Apel, Spartan Micro, and Vastrax. MP works on the data safety monitoring board for Medtronic. CS has research support from Penumbra, and is a shareholder in Neurotechnology Investors. KW is a research consultant for Penumbra. OZ consults for Stryker, Medtronic, Penumbra, Cerenovus, has research support from Genentech, Stryker, Medtronic, Penumbra and Cerenovus, and is a founding member of Galaxy Therapeutics, Inc. AS is a consultant for Terumo, Penumbra, Stryker, Cerenovus, and has non-financial research support from RAPID IA.

## Publisher's Note

All claims expressed in this article are solely those of the authors and do not necessarily represent those of their affiliated organizations, or those of the publisher, the editors and the reviewers. Any product that may be evaluated in this article, or claim that may be made by its manufacturer, is not guaranteed or endorsed by the publisher.
