# Supplementary material for: Safety and Long-Term Efficacy Outcomes for Endovascular Treatment of Wide-Neck Bifurcation Aneurysms of the Middle Cerebral Artery: Insights From the SMART Registry
Source: Front Neurol. 2022 Feb 7;13:830296. doi: 10.3389/fneur.2022.830296 (PMC8860028; doi:10.3389/fneur.2022.830296)
Supplement: Supplementary file 1 [file Data_Sheet_1.docx]

| **Site Count** | **IRB Chairperson (Name & Address)** |
| --- | --- |
| 1 | David Markenson, HCA HealthOne IRB  4900 South Monaco Street Suite 220 Denver, Colorado 80237 |
| 2 | Richard Stevenson  University of Virginia IRB for Health Sciences  Research  P.O.PO Box 800483  Charlottesville, VA Virginia 22908 |
| 3 | Harold Carlson  Stony Brook University IRB  W5530 Frank Melville Jr. Memorial Library  Stony Brook, NY 11794-3368  Stony Brook University Research IRB |
| 4 | Joy Cavagnaro  Troy Priest  Mitchell Reddish  Anita Tarzian  6940 Columbia Gateway Dr., Suite 110, Columbia, MC 21046 |
| 5 | Bert Wilkins  Western Institutional Review Board  [WIRB Panel 02]  1019 39^th^ Ave SE / Suite 120  Puyallup, WA 98374 |
| 6 | Henry K. Driscoll  Office of Research Integrity  One Marshall Drive, Huntington, WV 25755 |
| 7 | Jeffrey Skiles  Valley Health/ Winchester Medical Center IRB, PO Box 3340, VA 22604 |
| 8 | Andrew Hardie  Office of Research Integrity  19 Hagood Avenue, HarborviewOffice Tower, Suite Mail Code A115, Room 1140, PO Box 855, Charleston, SC |
| 9 | Allen Smith, MD  Covenant Health IRB  280 Fort Sanders West Boulevard  Bldg 4, Suite 204, Knoxville, TN 37922 |
| 10 | Undisclosed chairperson name  CHI Institute Research and Innovation  198 Inverness Dr W, Englewood, CO 80112 |
| 11 | Florencia Que, MD (co-chair, signer of initial IRB approval)  Eugene Kwon, MD (co-chair)  Mayo Clinic IRB  200 First St. SW  Rochester, MN 55905 |
| 12 | Theodore Bania, MD  Mount Sinai School of Medicine IRB #1 – Board B  One Gustave L. Levy Place Box 1081  345 E. 102^nd^ St., Suite 200  New York, NY 10029 |
| 13 | Michael Cimino  University at Buffalo IRB  875 Ellicott Street, room 5018  Buffalo, NY 14203 |
| 14 | Dr. Phillip T. Neff  SCL Health  1375 East 19th Avenue  Denver, CO  80218 |
| 15 | Dr. Manoo Bhakta  Chattanooga Unit of the College of Medicine IRB  960 East Third St.  Suite 100  Chattanooga, TN 37403 |
| 16 | Bert Wilkins  Western Institutional Review Board  1019 39^th^ Ave SE / Suite 120  Puyallup, WA 98374 |
| 17 | Wilkins Bert (Raymond)JD, MHA  Western Institutional Review Board  1019 39^th^ Avenue SE Suite 120  Puyallup, WA 98374 |
| 18 | Lamya Jarjour, MD (IRB Co-Chair)  Jeffrey Braff, DrPH (IRB Co-Chair)  Dignity Health IRB  3400 Data Drive  Rancho Cordova, California 95670 |
| 19 | Wilkins Bert (Raymond)JD, MHA  Western Institutional Review Board  1019 39^th^ Avenue SE Suite 120  Puyallup, WA 98374 |
| 20 | Wilkins Bert (Raymond)JD, MHA  Western Institutional Review Board  1019 39^th^ Avenue SE Suite 120  Puyallup, WA 98374 |
| 21 | Chair not Disclosed (Helen F. Graham Cancer Center & Resesarch Center  West Pavilion – Suite 2350  4701 Ogletown Stanton Road  Newark, DE 19713) |
| 22 | Karen Beckman  1105 N. Stonewall Avenue  Oklahoma City, OK 73117 |
| 23 | Bert Wilkins  Western Institutional Review Board  [WIRB Panel 02]  1019 39^th^ Ave SE / Suite 120  Puyallup, WA 98374 |
| 24 | Thomas Harter  Gundersen Clinic, Ltd.  1836 South Ave.  La Crosse, WI 54601 |
| 25 | Ronald Harris  UHSH Office of Clinical Trials  33-57 Harrison St.  Johnson City, NY 13790 |
| 26 | H. Lester Kircher and William E. Crowder Jr.  Geisinger Medical Center  100 N. Academy Ave.  Danville, PA 17822 |
| 27 | Krishna Rao  Springfield Committee for Research Involving Human Subjects  201 E. Madison St.  PO Box 19664  Springfield, IL 62794 |
| 28 | Vikas Desai  MetroWest Medical Center IRB  115 Lincoln St.  Framingham, MA 01702 |
| 29 | Mercy Health North LLC  Research Oversight and Education  2200 Jefferson Ave.  Toledo, Ohio 43604 |
| 30 | Bert Wilkins  Western Institutional Review Board  [WIRB Panel 02]  1019 39^th^ Ave SE / Suite 120  Puyallup, WA 98374 |
| 31 | Bert Wilkins  Western Institutional Review Board  [WIRB Panel 02]  1019 39^th^ Ave SE / Suite 120  Puyallup, WA 98374 |
